# Estimands for Clinical Effectiveness of Risk-Reducing Early Salpingectomy in Women With High Risk of Ovarian Cancer

**DOI:** 10.1001/jamanetworkopen.2025.32195

**Published:** 2025-09-16

**Authors:** Jacqueline Sia, Emily F. Lane, Caitlin T. Fierheller, Samuel Oxley, Ashwin Kalra, Michail Sideris, Xia Wei, Lea Mansour, Annika Idahl, Holly Fraser, Subhasheenee Ganesan, Priyanka Deshmukh, Raji Ganesan, Helen Hanson, Ertan Saridogan, Hisham Hamed, W Glenn McCluggage, Rosa Legood, Peter Sasieni, D Gareth Evans, Usha Menon, Adam Brentnall, Ranjit Manchanda

**Affiliations:** 1 Wolfson Institute of Population Health, Cancer Research UK, Barts Centre, Queen Mary University of London, London, United Kingdom; 2 Department of Gynaecological Oncology, Royal London Hospital, Barts Health NHS Trust, London, United Kingdom; 3 Department of Health Services Research and Policy, London School of Hygiene and Tropical Medicine, London, United Kingdom; 4 Department of Clinical Sciences, Obstetrics and Gynecology, Umeå University, Umeå, Sweden; 5 Birmingham Women’s Hospital, Birmingham Women’s and Children’s National Health Service (NHS) Foundation Trust, Birmingham, United Kingdom; 6 Peninsula Clinical Genetics, Royal Devon University Healthcare NHS Foundation Trust, Exeter, United Kingdom; 7 Faculty of Health and Life Sciences, University of Exeter Medical School, Exeter, United Kingdom; 8 University College London Hospitals NHS Foundation Trust, London, United Kingdom; 9 Guy’s and St Thomas’ NHS Foundation Trust, London, United Kingdom; 10 Department of Pathology, Belfast Health and Social Care Trust, Belfast, United Kingdom; 11 Manchester Centre for Genomic Medicine, Division of Evolution, Infection and Genomic Sciences, University of Manchester, Manchester Academic Health Science Centre, Manchester, United Kingdom; 12 Medical Research Council Clinical Trials Unit at University College London, Institute of Clinical Trials and Methodology, Faculty of Population Health Sciences, University College London, London, United Kingdom

## Abstract

**Importance:**

Risk-reducing early-salpingectomy (RRES) and delayed oophorectomy (DO) is a novel 2-stage alternative prevention strategy to risk-reducing salpingo-oophorectomy (RRSO) that avoids detrimental consequences of premature menopause. However, direct data on the clinical effectiveness for ovarian cancer (OC) risk reduction are lacking.

**Objective:**

To explore how to define clinical effectiveness from prospective cohort studies using the estimand framework and sample size requirements.

**Design, Setting, and Participants:**

In this comparative effectiveness research study, estimand and analysis options were considered to evaluate the clinical effectiveness of RRES with DO by extending the UK PROTECTOR cohort study, a multicenter, prospective, observational, national cohort study (N = 1250 recruited from January 1, 2019, to December 31, 2024) evaluating RRES and DO for OC surgical prevention. Eligibility criteria for participants were broadly premenopausal women 30 years or older at increased OC risk due to *BRCA1/BRCA2* pathogenic variants. Participants could choose RRES, RRSO, or no surgery at entry. Sample-size requirements to extend recruitment used initial data (eg, age and *BRCA1/BRCA2* distribution) from PROTECTOR (analysis undertaken from January 1, 2024, to December 31, 2025).

**Main Outcomes and Measures:**

Incidence of OC after (not at) RRES and before or at DO in women with normal histologic analysis findings at surgery. The proportion of cancers prevented was estimated as the completement of the observed (O) to expected (E; assuming no preventive effect of surgery) number of cancers detected (1 – O/E).

**Results:**

Initial data were obtained from 889 women in PROTECTOR (overall mean [SD] age, 39 [4.9] years), with 255 (28.7%) choosing RRSO (mean [SD] age, 42 [4.4] years), 405 (45.5%) choosing RRES (mean [SD] age, 38 [4.4] years), and 229 (25.7%) choosing no surgery (mean [SD], 38 [4.6] years). The preferred estimand outcome was OC incidence after surgery (RRES or RRSO) with a “while on intervention” strategy to account for intercurrent events. The primary target measure was the proportion of cancers prevented for RRES vs no surgery with superiority testing. A secondary target measure was noninferiority of RRES vs RRSO. An estimated 1150 RRES participants with 8 to 10 years of follow-up would provide approximately 92% power to show that 20% or more of cancers are prevented using a 1-sample binomial test of the O:E risk (external reference) at the 5% level under a range of assumptions and at least the same power for a noninferiority margin for the proportion of cancers prevented by RRES of those prevented by RRSO. Estimands based on incidence ratios had an infeasible sample size.

**Conclusions and Relevance:**

For this comparative effectiveness study of UK *BRCA* carriers, the recommended estimand differed from other ongoing clinical-effectiveness studies of RRES and DO. Advantages include direct use of expected risk at baseline (unknown at design stage), easier interpretation across cohorts than absolute risk differences, and providing a feasible recruitment target for PROTECTOR to evaluate clinical effectiveness.

## Key Points

**Question:**

What estimands are most useful for the study of clinical effectiveness of risk-reducing early salpingectomy (RRES) from prospective cohort studies?

**Findings:**

This comparative effectiveness study of 889 women used estimand and analysis options to evaluate clinical effectiveness of RRES with delayed oophorectomy, using initial data from the PROTECTOR cohort study. The preferred estimand outcome was ovarian cancer incidence after surgery, and the primary target measure was proportion of cancers prevented by RRES vs no surgery with superiority testing.

**Meaning:**

This estimand differs from those in other ongoing clinical effectiveness studies and suggests a feasible recruitment target for evaluating clinical effectiveness in the PROTECTOR cohort study.

## Teaser

This comparative effectiveness research study considers how to define clinical effectiveness from prospective cohort studies of women undergoing early salpingectomy with delayed oophortectomy vs salpingo-oophorectomy to reduce the risk of ovarian cancer using the estimand framework and how to evaluate sample size requirements.

## Introduction

Every year approximately 200 000 women die from ovarian cancer (OC) worldwide.^1^ Tubo-ovarian high-grade serous carcinoma (HGSC) is the most common histologic type (70%-80%), with very poor survival rates when detected at an advanced stage.^2^ Approximately 1 in 5 HGSCs are associated with pathogenic variants (PVs) in high-risk (eg, *BRCA1 *[OMIM 113705] and* BRCA2 *[OMIM 600185) and moderate-risk (eg, *RAD51C *[OMIM 602774]*, RAD51D *[OMIM 602954]*, BRIP1 *[OMIM 605882]*, *and* PALB2 *[OMIM 610355]) cancer susceptibility genes and are potentially preventable.^3,4^ *BRCA1 *and* BRCA2* PV carriers have an approximate 44% and 17% lifetime OC risk, respectively.^5^ Risk-reducing salpingo-oophorectomy (RRSO) is the most effective option for preventing OC. RRSO substantially reduces OC risk,^6,7^ along-with all-cause and OC-specific mortality in *BRCA* PV carriers.^8^ It is cost-effective^3^ and recommended by clinical guidelines^9,10^ for those 35 years and older for *BRCA1* PV carriers and 40 years and older for *BRCA2* PV carriers.^3,9^

Premenopausal RRSO causes premature surgical menopause and thus potential harms to long-term health, including increased risk of heart disease,^11^ osteoporosis, vasomotor symptoms, mood or sleep disturbance, reduced libido, vaginal dryness, sexual dysfunction, and neurocognitive decline, particularly if hormone replacement therapy use is not possible.^12,13,14,15^ Furthermore, hormone replacement therapy does not fully alleviate vasomotor symptoms or sexual dysfunction, with symptom levels remaining above those retaining their ovaries. Higher regret rates occur with premenopausal (approximately 10%) vs postmenopausal (1%) RRSO.^16^ Many women choose to delay RRSO until after natural menopause due to these potential harms.

There is compelling evidence and broad acceptance that most HGSCs originate from the fallopian tube.^17^ Serous tubal intraepithelial carcinoma (STIC) is established as a precursor lesion to OC.^18,19^ This has led to interest in risk-reducing early salpingectomy (RRES) followed by delayed oophorectomy (DO) as an alternative surgical prevention strategy to RRSO in women at high risk of familial OC,^12^ which can avoid detrimental effects from early menopause.

Studies have found that RRES and DO (RRESDO) is associated with high acceptability,^16^ satisfaction,^20^ and decreased cancer worry^21^ among premenopausal high-risk women and high clinical acceptability.^22^ Women concerned about sexual dysfunction are more likely to find RRESDO acceptable.^16^ Prospective observational studies (PROTECTOR^23^ and TUBA^24^) initially addressed outcomes of sexual function, menopause symptoms, and quality of life. Women underoing early salpingectomy have improved sexual function and menopause symptoms compared with RRSO.^25^ Decision-making is a complex process, which changes over time and may be influenced by multiple factors. Women choosing RRES may place a higher priority on menopause-related quality of life, concerns on impact on sexual function, or need to mitigate other competing risks and accept a likely lower OC risk reduction until DO, while women preferring RRSO may prioritize OC risk reduction.^16,20,26,27,28^

A remaining knowledge gap is the clinical effectiveness of early salpingectomy (ie, how much the operation reduces OC risk). Direct evidence on clinical effectiveness is important for policymakers for recommending RRESDO for routine clinical practice, informed counseling and surgical decision-making by patients, cost-effectiveness analysis, and guideline development. Randomized clinical trials are unacceptable to patients and clinicians and unethical, so observational evidence is needed.

Ongoing, prospective, observational studies are following up participants who choose among different surgical prevention options. These studies include TUBA-WISP-II (US and the Netherlands),^29^ SOROCk (US),^30^ and PROTECTOR (UK).^23^ The initial focus of the TUBA and PROTECTOR cohorts was the impact of RRESDO on sexual function, hormone levels, quality of life, and overall satisfaction.^25^ Because of the benefits observed on these measures, there is now interest in evaluating clinical effectiveness of RRES and DO by extending cohort recruitment and follow-up. In this article, we consider how to define clinical effectiveness from prospective cohort studies using the estimand framework^31^ and evaluate sample size requirements.

## Methods

### Design

**Figure 1.**
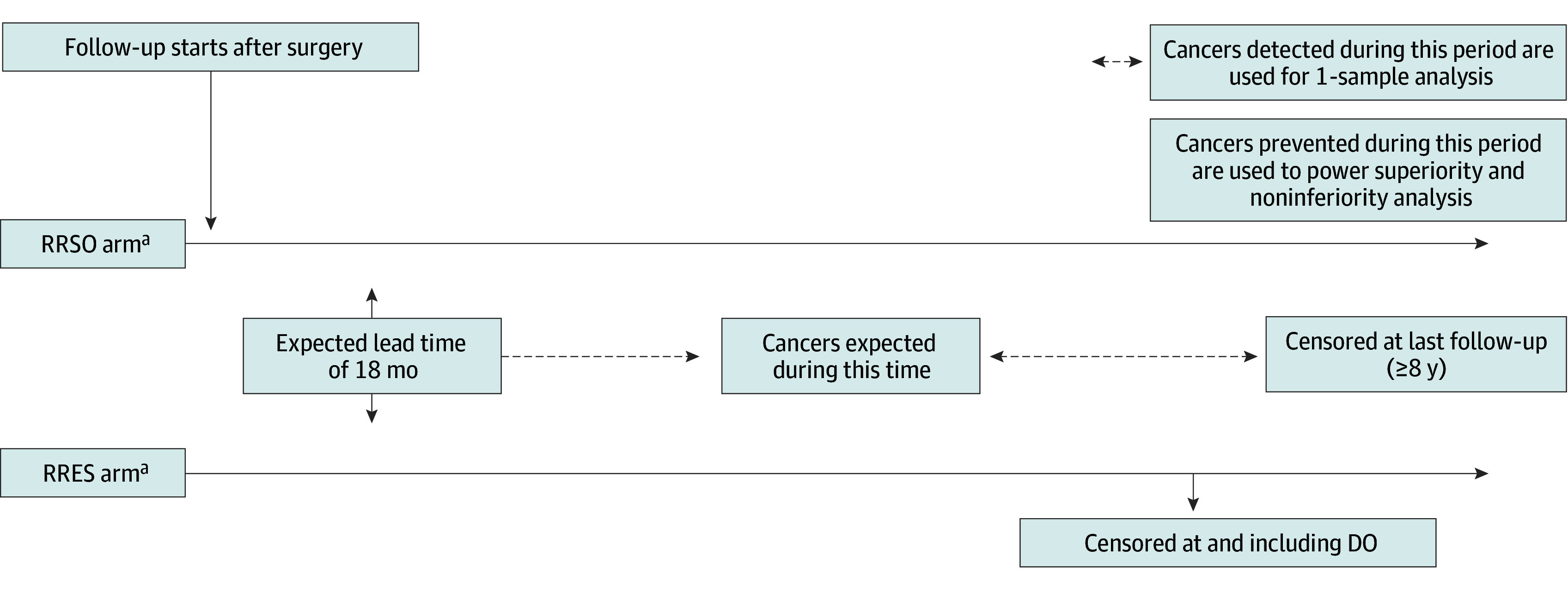
Population, Interventions, Outcome, and Intercurrent Event Treatment in PROTECTOR-2 DO indicates delayed oophorectomy; RRES, risk-reducing early salpingectomy; RRSO, risk-reducing salpingo-oophorectomy. ^a^Occult cancers at surgery (RRES or RRSO) are excluded from analysis.

PROTECTOR^23^ is a multicenter, prospective, observational, national cohort study evaluating RRESDO for surgical prevention of OC (N = 1250 recruited from January 1, 2019, to December 31, 2024). Participants choose among 3 study arms: RRESDO, RRSO, or no surgery (control). Control participants may later opt for surgical intervention. Participants in the RRES arm undergo DO later at a time of their choosing or at menopause, so the entire strategy is RRES with DO. The PROTECTOR study received approval from the Bloomsbury Research Ethics Committee. This analysis is covered within ethical approval obtained for data analysis using PROTECTOR data. All PROTECTOR participants provided written informed consent for providing data, study participation, and use of data for analysis. Recruitment rates of 25 to 30 participants per month have been achieved in PROTECTOR. An extension to PROTECTOR (PROTECTOR-2) aims to evaluate the clinical effectiveness of RRES for OC prevention in high-risk women compared with no surgery. We aim to estimate the OC risk reduction between RRES and DO (Figure 1). We followed the recommendations of the International Society for Pharmacoeconomics and Outcomes Research (ISPOR) reporting guideline for comparative effectiveness research.^32^ As recommended, we a priori specifed the research question, used a structured abstract for reporting, used a structured and standardized approach for analysis and reporting in terms of an estimand framework, and provided the source code for our analysis for enhancing reproducibility.

### Statistical Methods

#### Preferred Estimands

To evaluate clinical effectiveness in a prospective study with the design as in PROTECTOR, we used the estimand framework.^31^ This required definition of the following components: target population; intervention; outcome; intercurrent events, comparators, framework, and measures. Our preferred values of these components are: (1) target population: premenopausal women 30 years or older at high risk of OC, with no future fertility wishes (completed family) (PROTECTOR eligibility)^23^; (2) intervention: RRES and subsequent DO (time of patient’s choosing), with recommendation to undergo DO by time of natural menopause; (3) outcome: incidence of OC; we chose this outcome because the aim of the surgical procedure is to reduce risk of OC; and (4) intercurrent events: a “while on intervention” strategy to account for intercurrent events; that is, for the RRES intervention group, the focus is the risk of OC after RRES until DO is completed; we chose “while on intervention” because the main clinical question is risk during the period after surgery and up to DO; timing of DO will depend on each woman’s decision, which is hard to anticipate; to date, unavailability and loss to follow-up for other reasons has been very low; events related to breast cancer (BC) diagnosis are unlikely to have a significant impact on OC risk. Finally, for (5) comparators, framework, and measures, the primary outcome is superiority of RRES vs no surgery based on the proportion of OC prevented. In the target population during a given follow-up, this proportion is defined as *R* = 1 – (*p*_s_/*p*_e_), where *p*_e_ is expected incidence without surgery and *p*_s_ is the (observed) incidence after surgery. To show superiority, we require *R* > 0.2. The margin provides assurance that RRES is at least moderately clinically effective in that it is associated with a risk reduction of at least 20%. This measure is particularly important for informed and balanced decision-making by individuals who find RRSO unacceptable. It has previously been used to evaluate utility of agents to prevent HIV and may lead to much smaller sample size requirements than incidence-based measures.^33^

A secondary outcome is noninferiority of RRES vs RRSO. Defining R_1_ and R_2_ as the proportion prevented in the RRES and RRSO groups, respectively, our summary measure is *S* = *R*_1_/*R*_2_ (ie, the proportion of OCs preventable by RRSO that are also preventable by RRES). To show noninferiority, we require this ratio to be greater than 0.2. This measure is used as a secondary outcome because it has relevance for those who might consider either surgical option, noting that no clinical or biological rationale exists for superiority of RRES compared with RRSO. RRES and DO may be preferred given major negative health impacts from early menopause, particularly if hormone replacement therapy is infeasible.

Another secondary outcome is absolute risk of OC after RRES. Direct evidence on risk of OC after surgery is important for decision-making, irrespective of whether it is less than without surgery.

#### Alternative Estimands

Other studies have used different estimands. These focus on noninferiority. They test whether the absolute-risk difference between RRES and RRSO is less than a prespecified margin.

The TUBA-WISP-II study focuses on noninferiority of RRES vs RRSO at or after surgery until DO.^29^ An important difference of this endpoint from our preference, is the inclusion of occult cancers detected at surgery in both groups.

The SOROCk study uses noninferiority of RRES vs RRSO for lifetime risk of OC after surgery.^30^ An important difference is that events are included after DO.

### Statistical Analysis

We first evaluated sample size requirements of our preferred estimand under a range of parameter scenarios (analysis undertaken from January 1, 2024, to December 31, 2025). R was estimated as the complement of the observed (O) to expected (E) number of cancers detected (1 – O/E). We estimated E using data from a meta-analysis^34^ (eTable 1 in Supplement 1) and early data from PROTECTOR on the age and *BRCA1 *and* BRCA2* distribution in those who consented to surgery. To evaluate sensitivity to expected risk assumptions, we also used lower incidence assumptions from the CanRisk and BOADICEA model (eTable 1 in Supplement 1).^35,36^ To account for lead time due to surgery at the start of the follow-up period, expected incidence excluded the first 1.5 years after surgery (excluded tubo-ovarian cancers detected at surgery). We considered power when increasing follow-up from 8 years on all participants until complete follow-up at 52 years of age for all (assumed to be the age at menopause for sample size estimation purposes). For effectiveness, we assumed a relative risk of 0.35 (65% risk reduction) for RRES vs no surgery. This was because approximately 70% of occult cancers identified in women with *BRCA* undergoing risk-reducing surgery are found in the tube and not the ovary,^37^ and an approximately 65% OC risk reduction from bilateral salpingectomy has been reported in low-risk women.^38^ Our sensitivity analysis also considered a 55% and 60% risk reduction for RRES. For RRSO vs no surgery, we assumed a 96% risk reduction, following reports of an approximately 97% risk reduction in UK women with *BRCA *PVs^6^ and an approximately 96% risk reduction in average-risk women after salpingo-oophorectomy.^39^ Together, these assumptions imply the expected ratio of OCs prevented by RRES to RRSO is 65:96 (68%). Sample size was estimated for 90% power at a 2-sided 5% level, allowing for a 5% dropout rate. The power assumes that Wilson 95% CI will be used for the primary estimand and a nonparametric empirical bootstrap 95% CI (5000 replications) for the secondary noninferiority comparison (between the RRES vs RRSO groups). Statistical computations were performed using R software, version R-4.4.3 (R Foundation for Statistical Computing) (see eMethods in Supplement 1 for the source code).^40^ Differences between primary estimands for clinical effectiveness in PROTECTOR with those planned for TUBA-WISP-II^29^ and SOROCk^30^ were analyzed.

## Results

Initial data were obtained from 889 women in PROTECTOR (overall mean [SD] age, 39 [5] years), with 255 (28.7%) choosing RRSO (mean [SD] age, 42 [4.4] years), 405 (45.5%) choosing RRES (mean [SD] age, 38 [4.4] years), and 229 (25.7%) choosing no surgery (mean [SD], 38 [4.6] years). The age as well as the *BRCA1 *and* BRCA2* distribution of participants at surgery are provided in eTable 2 in Supplement 1 (441 [49.6%] with *BRCA1* and 448 [50.4%] with *BRCA2*). eTable 2 in Supplement 1 also details the expected incidence (without surgery) for 8- to 10-year follow-up (or to 52 years of age) from the 2 incidence assumptions. For example, using the main assumption, a *BRCA1* carrier aged 40 years had a 7.3% expected risk after surgery if it is ineffective (1.6% for a *BRCA2* carrier).

In the base scenario with 8-year follow-up, the expected risks were 3.0% in the RRES arm and 4.4% in the RRSO arm. Assuming no intercurrent events (other than DO), using the age as well as *BRCA1 *and* BRCA2* distribution to date in PROTECTOR, and using other assumptions discussed below (eBox in Supplement 1), we estimated that 1150 participants with 8-year follow-up in the RRES arm would provide approximately 92% power to determine that 20% or more of OC is prevented (ie, an absolute OC risk reduction from 3% to <2.4% or expected events from 35 to <28, assuming 12 expected with RRES). We judge that this is sufficiently precise to evaluate the likely efficacy of RRESDO vs control (no surgery). Correspondingly, there would be approximately 90% power to determine the proportion of OCs prevented by RRSO (700 RRSO participants; absolute risk, 1.2%) that is achieved by RRES is at least 28% (secondary outcome)(eTable 4 in Supplement 1). Implications for recruitment to PROTECTOR are shown in Figure 1.

Sensitivity analysis using a lower expected cancer incidence with BOADICEA assumptions showed slightly reduced power, such as from 92% to 83% for the main scenario with a sample size of 1150 (eTable 3 in Supplement 1). Power was also sensitive to the assumption of the efficacy of RRES being approximately 12% less if it was 60% vs 65% (eTable 3 in Supplement 1). If 1000 participants were recruited, then power in the main scenario decreased from 92% to 86%. One way to mitigate the potential effects of these factors in the study is to monitor recruitment, expected risk, and delay analysis until the expected number of events (without surgery) is sufficiently high to ensure adequate power (eTable 3 in Supplement 1).

**Figure 2.**
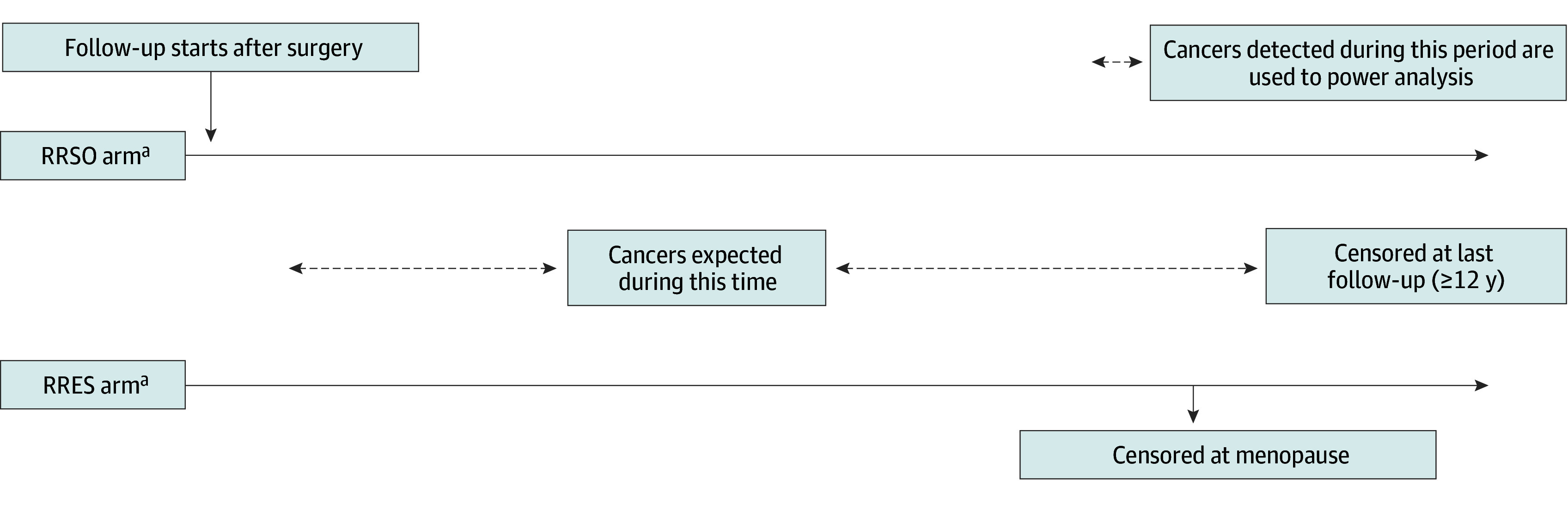
Noninferiority of Risk-Reducing Early Salpingectomy (RRES) vs Risk-Reducing Salpingo-Oophorectomy (RRSO) for Ovarian Cancer Incidence After Surgery and Until and Including Delayed Oophorectomy ^a^Occult cancers at surgery (RRES or RRSO) are excluded from analysis.

A noninferiority evaluation of OC incidence after RRES vs RRSO after surgery (and until and including DO) (Figure 2) would require a much larger sample size under the same assumptions. For example, if a relative noninferiority margin of 20 (0.04 × 20 = 0.8 relative risk vs no surgery) is used, we estimated the need to recruit 11 700 participants into the RRESDO arm with 12-years follow-up for 90% power (total sample size including RRSO arm being 19 200). Although this estimand does not address risk after DO, 5-year additional follow-up after DO would provide approximately 90% power to test that a reduction in OC risk is more than 70%, vs no surgery in a cohort of 1150 women with 10% dropout (eFigure 1 in Supplement 1).

### Comparison of Estimands in TUBA-WISP-II and SOROCk 

**Figure 3.**
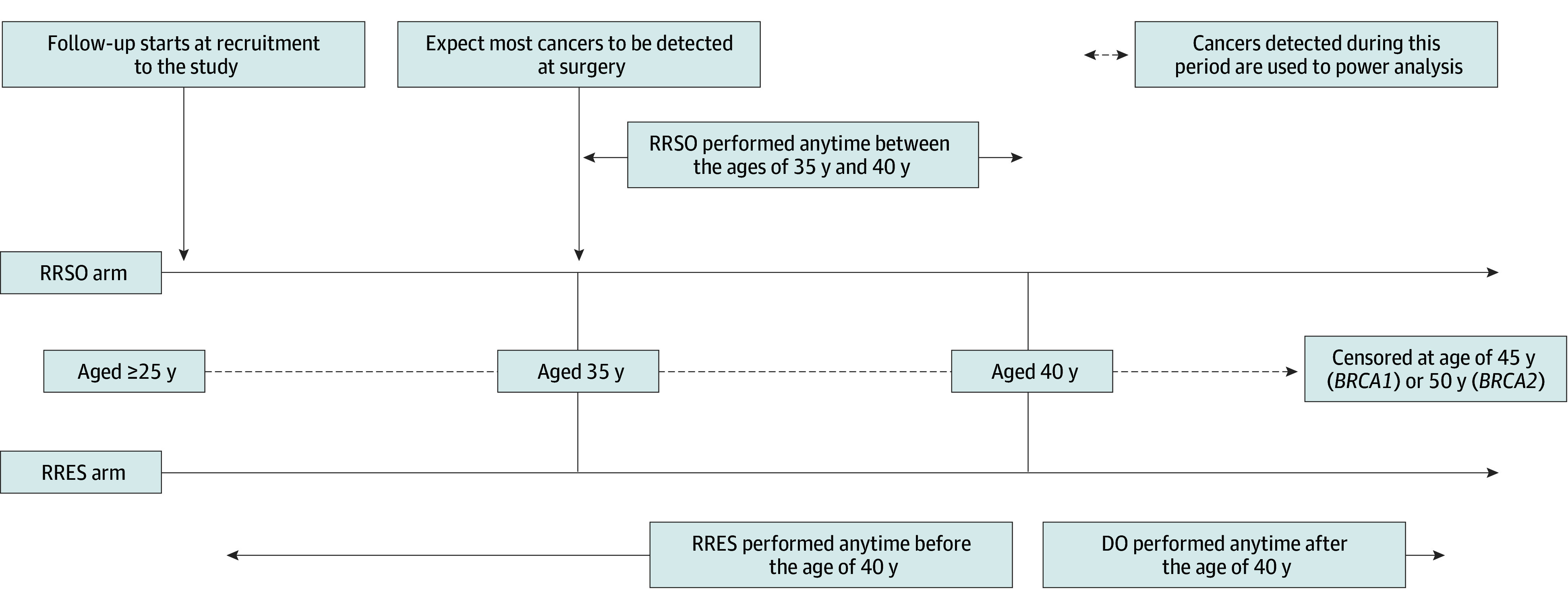
Noninferiority of Risk-Reducing Early Salpingectomy (RRES) vs Risk-Reducing Salpingo-Oophorectomy (RRSO) for Ovarian Cancer Incidence at or After Surgery Until Delayed Oophorectomy (DO) (TUBA-WISP-II)

The TUBA-WISP-II (n = 3000) estimand for clinical effectiveness has a different population, approach to intercurrent events, and testing framework (Table, Figure 3). TUBA-WISP-II evaluates follow-up before surgery and includes cancers detected at surgery (for which we expect a similar detection rate regardless of RRES or RRSO). TUBA-WISP-II evaluates whether absolute risk differs by more than 2% between the RRESDO and RRSO groups. In TUBA-WISP-II, most events are likely occult cancers as identified by histologic analysis after surgery and not related to any risk reduction benefit. OCs diagnosed at the time of risk-reducing surgery, before surgery has had any effect, and after RRSO or DO, when incidence might be expected to be the same, are included in the noninferiority comparison. In other words, any difference in risk after RRES surgery and before DO may be masked by those before RRES, at RRES, and after DO, when we would not expect differences. A noninferior result with this design may not mean that this is true after RRES surgery and before DO.

**Table.** Estimands Framework for TUBA-WISP-II, SOROCk, and PROTECTOR-2

Variable	Noninferiority of RRES vs RRSO at or after surgery until DO (TUBA-WISP-II)^29^	Noninferiority of RRES vs RRSO for lifetime risk after surgery (SOROCk)^30^	1-Sample analysis of RRES vs no surgery after RRES and up to and including DO (PROTECTOR-2)
Population inclusion criteria			
Menopausal status	Premenopausal women	Premenopausal or postmenopausal women	Premenopausal women
Germline PV	*BRCA1, BRCA2, RAD51C, RAD51D, BRIP1*	*BRCA1*	*BRCA1, BRCA2*
Age at inclusion	*BRCA1*: aged 25-40 y, *BRCA2*: aged 25-45 y, *RAD51C, RAD51D, *and* BRIP1*: aged 25-50 y	Aged 35-50 y	Aged >30 y
Surgical status	Presence of at least 1 fallopian tube	Presence of at least 1 ovary and fallopian tube	No previous bilateral salpingectomy or bilateral oophorectomy
Other	Childbearing completed, no personal history of nonovarian malignant tumor	No personal history of ovarian cancer	Childbearing completed, no personal history of tubal, ovarian, or peritoneal malignant tumor
Intervention	Bilateral salpingectomy with delayed oophorectomy	Bilateral salpingectomy with optional delayed oophorectomy	Bilateral salpingectomy
Comparator	Bilateral salpingo-oophorectomy	Bilateral salpingo-oophorectomy	No surgery or bilateral salpingo-oophorectomy
Outcome	Ovarian cancer incidence (from study entry until age of 45 y for *BRCA1* and 50 y for *BRCA2* PV carriers)	Ovarian, primary peritoneal, or fallopian tube cancer (20-year follow up)	Ovarian cancer incidence (8-year follow up)
Estimand summary (a description of the intervention effect the study aims to quantify)	Absolute difference in ovarian cancer incidence in women undergoing RRES with DO compared with women undergoing RRSO	Absolute difference of incident high-grade serous ovarian, primary peritoneal, or fallopian tube cancer in women undergoing RRES (and optional DO) compared with women undergoing RRSO	Proportion of ovarian cancers prevented compared with no surgery in women undergoing RRES during the period after surgery and up to and including DO and relative proportion of ovarian cancers prevented from RRES compared with RRSO corresponding to the period after surgery (RRES) and up to and including DO
Estimator (statistical method used to compute the effect measure)	The primary outcome of cumulative tubo-ovarian cancer incidence at the target age will be analyzed per *BRCA*-type using Kaplan-Meier analysis with stabilized IPTW and the stabilized IPTW weights, used to adjust for possible imbalances, will be defined based on a logistic regression model, using as the dependent variable the actual treatment and as independent variables possible confounders, that is, at least age at inclusion, history of breast cancer, family history of tubo-ovarian or breast cancer, and region (US, European Union, Australia); each observation will be weighted with its own stabilized IPTW given the observed values of the confounders	The absolute difference will be estimated using a proportional hazards regression analysis (Cox model) adjusted for familial history of gynecologic cancer and age at study entry	1-Sample analysis of the proportion of expected cancers (based on germline mutation, menopausal status, and age) observed in follow-up (95% CI using the Wilson method) and ratio of the proportion of cancers prevented in after RRES until DO compared with RRSO (95% CI using a nonparametric bootstrap); for both estimators, the expected cancer incidence without surgery is estimated using meta-analysis data on *BRCA1/2* penetrance by age
Analysis methods	Noninferiority is reached when the upper limit of the 1-sided 97.5% CI for the difference in cumulative incidence between salpingectomy with DO and salpingo-oophorectomy is ≤2.0% for *BRCA1* and ≤1.5% for *BRCA2* PV carriers; noninferiority margin for each *BRCA* group is a 2% risk difference	Noninferiority margin is 1% risk difference; estimated using a Cox proportional hazards regression model	Superiority margin is >20% prevention (ie, lower limit on a 2-sided 95% CI on the proportion of cancers prevented is >20%); noninferiority margin for the sample size as superiority testing is estimated at >27% of prevention achieved by RRSO is achieved by RRES; sensitivity analysis is used to evaluate robustness to these assumptions; number of cancers diagnosed in the control group will also provide a way to verify gross departures from the assumption

Abbreviations: DO, delayed oophorectomy; IPTW, inverse probability of treatment weighting; PV, pathogenic variant; RRES, risk-reducing early salpingectomy; RRSO, risk-reducing salpingo-oophorectomy.

**Figure 4.**
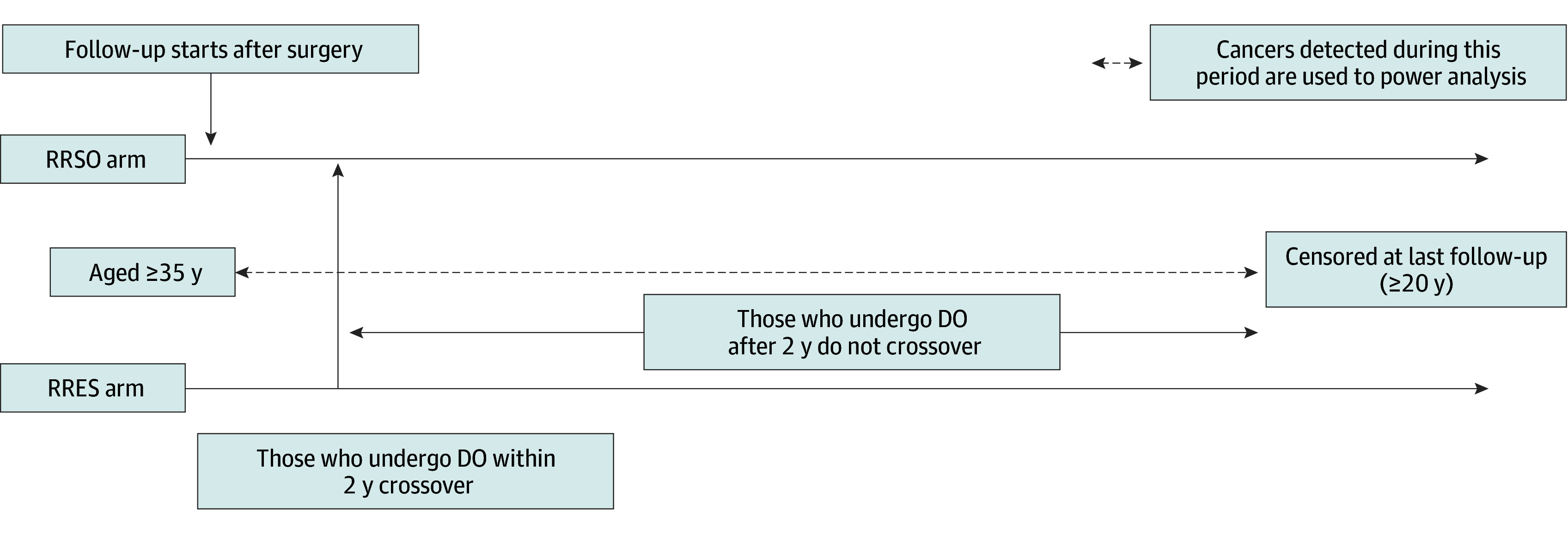
Noninferiority of Risk-Reducing Early Salpingectomy (RRES) vs Risk-Reducing Salpingo-Oophorectomy (RRSO) for Lifetime Ovarian Cancer Risk After Surgery (SOROCk) DO indicates delayed oophorectomy.

The estimand from SOROCk (n = 2262) differs in terms of intercurrent events and testing framework (Table and Figure 4). The study will evaluate residual lifetime risk for all participants (ie, including after the second DO operation). SOROCk also includes postmenopausal women, which is different from recommended UK practice. The summary measure is an absolute risk difference for which equal effectiveness and a 1% margin are assumed in their sample size calculation. We do not believe equal effectiveness is a reasonable assumption and therefore cannot justify using the same margin for an extension to PROTECTOR. For example, if expected risk is greater than 1/(0.35 − 0.04) = 3.2%, then there will be a (true) greater than 1% absolute risk difference. Additionally, the study plans analysis based on accumulated events, with a sample size of 53 (2.3% of 2262) required for primary analysis. However, if events at surgery are excluded (as in the preferred estimand), then even if RRES and RRSO are equal with a 65% reduction in risk, an event rate of 2.3% in RRES would correspond to an expected rate of 6.6% without surgery, which would require a greater than 12-year follow-up under a range of scenarios (eTable 4 in Supplement 1).

## Discussion

We evaluated estimands for clinical effectiveness (OC risk reduction) of RRES and DO using data from the PROTECTOR study. Our preferred estimand outcome was ovarian cancer incidence after surgery, with the primary target measure being proportion of cancers prevented for RRES vs no-surgery with superiority testing. Our preferred estimand and method for analysis allow an achievable recruitment target and a relatively shorter follow-up period in a UK setting before reporting results on clinical effectiveness. One alternative estimand measure, which we do not recommend, is the incidence ratio. We believe this is not useful for prevention interventions because it requires an infeasibly large sample size for precision due to a small number of events expected. For example, at an extreme with 2 interventions that prevent 100% of OCs, incidence would be 0% for both. Therefore, even with a very large sample size, a study would not be able to estimate the risk ratio with precision.

Two other ongoing studies, TUBA-WISP-II^29^ (Figure 3) and SOROCk^30^ (Figure 4), use a noninferiority estimand^31^ based on an absolute risk difference (Table). We have not included this directly because the ratio of cancers prevented can be reexpressed in terms of absolute risk difference, if desired. Potential reasons for preferring the ratio of cancers prevented (compared with absolute risk difference) include that the (implicit) absolute risk margin adapts if expected risk is different than anticipated at design stage. This might occur if, for example, follow-up from participants is different, such as due to earlier DO or there are differences from assumed age and *BRCA* distribution in the cohort, or because of errors in assumptions on true efficacy. For example, if the expected risk without surgery is lower at analysis than anticipated at design stage then a smaller margin may be needed to ensure noninferiority is not declared when risk after RRES may be equivalent to no surgery. Focus on the proportion of cancers prevented avoids the need to change noninferiority absolute risk margins post hoc.

This study’s findings are anticipated to be especially important in an era of increasing awareness, acceptability of, and access to genetic testing, along-with expanding guidelines and policy as well as pathways leading to increased identification of high-risk women who can benefit from RRES and DO. Examples include increased testing at cancer diagnosis,^41^ Jewish population–based *BRCA* testing programs (UK and Israel),^42,43,44^ decreasing genetic testing thresholds (eg, new National Institute for Health and Care Excellence guidelines),^4,9^ and ongoing general population research studies for genetic testing of cancer susceptibility genes (eg, PROTECT-C).^45^

### Strengths and Limitations

This study has strengths. A strength of our preferred primary and secondary estimands is that they address the key issue of risk reduction from RRES during the most informative and important period between RRES and DO, which has the greatest potential difference between RRES and RRSO. Patient and public involvement in PROTECTOR identified this to be a key issue for participants and their decision-making and necessary for health economic evaluation. Individuals choosing RRES may accept a lower level of risk reduction (rather than doing nothing) as a tradeoff for avoiding negative impact of early menopause.^20^ In addition, it is the magnitude of RRES compared with no surgery or RRSO that is most relevant to communicate to patients for informed decision-making.^31^ Future patient and public involvement work in PROTECTOR will assess what a minimum acceptable level of OC risk reduction with RRES would be for patients. A statistical strength of focusing on the proportion of cancers prevented as a summary measure is the potential interpretation across different cohorts (unlike absolute risk differences). We therefore believe it could be a worthwhile secondary outcome in other ongoing studies. Additionally, with our proposed estimand and analysis approach, PROTECTOR-2 is powered for noninferiority and superiority, and the margin for noninferiority is less stringent than for superiority (in contrast to incidence-based analysis). This benefit is a natural consequence of focusing on cancers prevented and using an external estimate of expected risk. Another benefit of incorporating expected risk into the estimand directly is that it provides an efficient method to determine when to report results. That is, rather than monitor events, one can monitor expected risk and therefore when to lock the database for primary analysis (ie, once this is >3%) in an adaptive manner (rather than, for example, relying on follow-up time reaching 8 years).

The study also has limitations. A limitation of our method for analysis is the need for a reliable estimate of expected OC risk without surgery. As such, a risk of bias is overestimation of the expected number of OCs for the superiority comparison, such as from an overrepresentation of (unaccounted) protective risk factors in the cohort. Analysis of the participants who do not select surgery in PROTECTOR will provide some evidence on gross departures from the expected risk. Furthermore, methods to adjust for different risk factors between the groups in an observational study will always be needed, so we do not view this as a major weakness of the method itself. Indeed, use of an external reference for expected risk is how secondary noninferiority analysis adjusts for differences in confounders between the RRES and RRSO groups. This framework might be made more robust by considering different model adjustments in a sensitivity analysis or by extending the analytical method to incorporate model uncertainty on the expected risk, such as through bayesian methods. Other methods to directly account for differences in risk between arms due to confounders using recorded confounders and outcomes (cancer incidence) from the study itself are likely to be unreliable due to a small number of expected events (eg, 3% × 0.35 × 1150 = 12 events expected in RRES at 8 years). Therefore, analysis that closely stratifies the arms by propensity scores or otherwise is likely to be of limited value because most strata will have zero events. Similarly, analysis based on direct adjustment for confounders through standard regression adjustments will not be reliable. Another limitation is that our estimand and analysis will not address risk after DO, but these factors might be evaluated subsequently. Although our estimand and analysis will not address risk after DO, this can be evaluated subsequently with 5 years of additional follow-up after DO, with 90% power for testing greater than 70% reduction in OC-risk (eFigure 1 in Supplement 1).

## Conclusions

Several international studies are evaluating the extent of OC risk reduction with RRESDO but with different estimands. Although each study will generate helpful data on the effect size of OC risk reduction to inform future policy or practice and enable informed decision-making for patients, we also think it would be important for investigators of the ongoing international studies to collaborate and undertake an individual patient data meta-analysis to harmonize analysis estimands. This approach will help to increase precision and understanding about the clinical effectiveness of RRES.

## Notes

Supplement 1. **eTable 1. **BRCA1 and BRCA2 penetrance assumptions used for sample size estimation, when using model A (based on Chen et al, 2020) and B (BOADICEA, 1980+ cohort) **eTable 2. **Assumptions used to estimate expected risk in PROTECTOR. The age and BRCA1/2 distribution is estimated initial recruits to PROTECTOR **eTable 3. **Estimated power for a one-sample O/E test given sample size of a preventive effect at least 20%, for different analysis time follow-up lengths, penetrance estimates (A= Chen et al (2020), B= Boadicea, 1908+ cohort; eTable 1), and effect sizes for RRES (e=0.65 is the main scenario, 0.60,0.55 as sensitivity analyses) **eTable 4. **Estimated non-inferiority threshold for 90% power testing that the cancers prevented between RRES and RRSO is at least 20%. Scenarios use different follow-up length (same for all), penetrance estimates (A= Chen et al (2020), B= BOADICEA, 1980+ cohort; eTable 1), and effect sizes for RRES (e=0.65 main scenario, 0.60,0.55 as sensitivity analyses) **eMethods. **Supplementary Methods **eFigure. **One-sample analysis of DO vs no surgery **eBox. **Sample size assumptions
eReferences

Supplement 2.
PROTECTOR Team Members

Supplement 3.
Data Sharing Statement
