# Errors in Abstract and Methods

**DOI:** 10.1001/jamanetworkopen.2025.42056

**Published:** 2025-10-08

In the Original Investigation titled “Estimands for Clinical Effectiveness of Risk-Reducing Early Salpingectomy in Women With High Risk of Ovarian Cancer,”^1^ published September 16, 2025, there were errors in how estimands were defined in the Abstract and Methods. This article has been corrected.^1^
