# Supplementary material for: Estimands for Clinical Effectiveness of Risk-Reducing Early Salpingectomy in Women With High Risk of Ovarian Cancer
Source: JAMA Netw Open. 2025 Sep 16;8(9):e2532195. doi: 10.1001/jamanetworkopen.2025.32195 (PMC12441877; doi:10.1001/jamanetworkopen.2025.32195)
Supplement: Supplement 1. — eTable 1. BRCA1 and BRCA2 penetrance assumptions used for sample size estimation, when using model A (based on Chen et al, 2020) and B (BOADICEA, 1980+ cohort) eTable 2. Assumptions used to estimate expected risk in PROTECTOR. The age and BRCA1/2 distribution is estimated initial recruits to PROTECTOR eTable 3. Estimated power for a one-sample O/E test given sample size of a preventive effect at least 20%, for different analysis time follow-up lengths, penetrance estimates (A= Chen et al (2020), B= Boadicea, 1908+ cohort; eTable 1), and effect sizes for RRES (e=0.65 is the main scenario, 0.60,0.55 as sensitivity analyses) eTable 4. Estimated non-inferiority threshold for 90% power testing that the cancers prevented between RRES and RRSO is at least 20%. Scenarios use different follow-up length (same for all), penetrance estimates (A= Chen et al (2020), B= BOADICEA, 1980+ cohort; eTable 1), and effect sizes for RRES (e=0.65 main scenario, 0.60,0.55 as sensitivity analyses) eMethods. Supplementary Methods eFigure. One-sample analysis of DO vs no surgery eBox. Sample size assumptions eReferences [file jamanetwopen-e2532195-s001.pdf]

Supplemental Online Content

Sia J, Lane EF, Fierheller C, et al Clinical effectiveness of risk-reducing early salpingectomy in high-risk women. *JAMA Netw Open*. 2025;8(9):e2532195. doi:10.1001/jamanetworkopen.2025.32195

eTable 1. BRCA1 and BRCA2 penetrance assumptions used for sample size estimation, when using model A (based on Chen et al, 2020) and B (BOADICEA, 1980+ cohort)
eTable 2. Assumptions used to estimate expected risk in PROTECTOR. The age and BRCA1/2 distribution is estimated initial recruits to PROTECTOR
eTable 3. Estimated power for a one-sample O/E test given sample size of a preventive effect at least 20%, for different analysis time follow-up lengths, penetrance estimates (A= Chen et al (2020), B= Boadicea, 1908+ cohort; eTable 1), and effect sizes for RRES (e=0.65 is the main scenario, 0.60,0.55 as sensitivity analyses)
eTable 4. Estimated non-inferiority threshold for 90% power testing that the cancers prevented between RRES and RRSO is at least 20%. Scenarios use different follow-up length (same for all), penetrance estimates (A= Chen et al (2020), B= BOADICEA, 1980+ cohort; eTable 1), and effect sizes for RRES (e=0.65 main scenario, 0.60,0.55 as sensitivity analyses)
eMethods. Supplementary Methods
eFigure. One-sample analysis of DO vs no surgery
eBox. Sample size assumptions
eReferences

This supplemental material has been provided by the authors to give readers additional information about their work.

© 2025 Sia J et al. *JAMA Network Open*.

eTable 1. BRCA1 and BRCA2 penetrance assumptions used for sample size estimation, when using model A (based on Chen et al, 2020) and B (BOADICEA, 1980+ cohort)

Age	BRCA1<br>A. Chen et al	BRCA1<br>B. Boadicea	BRCA2<br>A. Chen	BRCA2<br>B. Boadicea
30	0.01%	0.01%	0.01%	0.01%
31	0.05%	0.07%	0.03%	0.01%
32	0.14%	0.16%	0.07%	0.02%
33	0.28%	0.27%	0.14%	0.02%
34	0.46%	0.41%	0.24%	0.03%
35	0.68%	0.60%	0.35%	0.04%
36	0.95%	0.85%	0.49%	0.05%
37	1.27%	1.16%	0.65%	0.06%
38	1.62%	1.58%	0.83%	0.07%
39	2.02%	2.12%	1.04%	0.08%
40	2.47%	2.83%	1.27%	0.09%
41	3.03%	3.75%	1.50%	0.10%
42	3.71%	4.67%	1.73%	0.12%
43	4.51%	5.59%	1.96%	0.14%
44	5.43%	6.51%	2.20%	0.17%
45	6.45%	7.42%	2.44%	0.22%
46	7.58%	8.33%	2.68%	0.27%
47	8.81%	9.24%	2.93%	0.35%
48	10.14%	10.15%	3.17%	0.46%
49	11.56%	11.05%	3.42%	0.61%
50	13.07%	11.94%	3.67%	0.82%
51	13.94%	12.82%	3.94%	1.10%
52	14.84%	13.73%	4.30%	1.47%
53	15.77%	14.67%	4.79%	1.98%
54	16.72%	15.63%	5.45%	2.66%
55	17.69%	16.62%	6.33%	3.56%

eTable 2. Assumptions used to estimate expected risk in PROTECTOR. The age and BRCA1/2 distribution is estimated initial recruits to PROTECTOR

© 2025 Sia J et al. *JAMA Network Open*.

The 8/10y risk for BRCA1/2 carriers by age are estimated using two estimates of Penetrance (A= Chen et al (2020), B= BOADICEA 1980+ cohort).

Age surgery (y)	PROTECTOR				Assumptions							
			BRCA1	BRCA1	BRCA1	BRCA1	BRCA2	BRCA2	BRCA1	BRCA1	BRCA2	BRCA2
	RRES (%)	RRSO (%)	RRES (%)	RRSO (%)	Model A 8y	Model A 10y	Model A 8y	Model A 10y	Model B 8y	Model B 10y	Model B 8y	Model B 10y
30	7.2	2.0	52.4	75.0	1.54	2.40	0.78	1.22	1.48	2.75	0.05	0.07
31	3.5	0.0	30.0	-	1.84	2.87	0.93	1.40	1.93	3.61	0.06	0.08
32	3.5	2.0	70.0	100.0	2.13	3.42	1.08	1.55	2.53	4.44	0.06	0.09
33	6.9	0.0	55.0	-	2.51	4.05	1.21	1.69	3.32	5.25	0.07	0.11
34	6.9	3.0	40.0	50.0	2.96	4.76	1.32	1.81	4.06	6.01	0.07	0.13
35	7.9	3.0	69.6	66.7	3.50	5.55	1.41	1.90	4.75	6.70	0.09	0.16
36	9.7	2.5	39.3	80.0	4.12	6.43	1.48	1.97	5.35	7.32	0.11	0.21
37	9.0	3.0	50.0	33.3	4.83	7.38	1.53	2.03	5.84	7.83	0.14	0.28
38	9.7	8.1	35.7	81.3	5.61	8.42	1.56	2.06	6.19	8.19	0.19	0.38
39	9.7	13.1	60.7	61.5	6.43	9.50	1.58	2.09	6.35	8.36	0.26	0.52
40	8.6	13.6	60.0	40.7	7.26	10.58	1.60	2.11	6.39	8.41	0.35	0.71
41	5.2	7.6	26.7	46.7	8.08	10.81	1.61	2.15	6.43	8.45	0.49	0.97
42	3.5	8.6	30.0	29.4	8.91	10.96	1.63	2.29	6.47	8.52	0.67	1.33
43	4.5	7.1	38.5	42.9	8.89	9.94	1.67	2.05	6.50	7.55	0.91	1.29
44	2.8	8.6	25.0	29.4	8.79	8.79	1.80	1.80	6.56	6.56	1.24	1.24
45	0.3	8.6	0.0	52.9	7.51	7.51	1.56	1.56	5.57	5.57	1.17	1.17
46	1.0	3.0	0.0	33.3	6.10	6.10	1.30	1.30	4.57	4.57	1.08	1.08
47	0.3	3.0	100.0	66.7	4.57	4.57	1.05	1.05	3.57	3.57	0.94	0.94
48	0.0	3.0	-	16.7	2.92	2.92	0.79	0.79	2.56	2.56	0.77	0.77
49	0.0	0.0	-	-	1.55	1.55	0.52	0.52	1.55	1.55	0.52	0.52

RRES (%)- % of participants in that age group in PROTECTOR RRES group
RRSO (%)- % of participants in that age group in PROTECTOR RRSO group
BRCA1 RRES (%) – % BRCA1 carriers by age in RRES group
BRCA1 RRSO (%) – % BRCA1 carriers by age in RRSO group
Last eight columns describe predicted risk with 8 or 10 years follow up by gene and model

eTable 3. Estimated power for a one-sample O/E test given sample size of a preventive effect at least 20%, for different analysis time follow-up lengths, penetrance estimates (A= Chen et al (2020), B= Boadicea, 1908+ cohort; eTable 1), and effect sizes for RRES (e=0.65 is the main scenario, 0.60,0.55 as sensitivity analyses)

© 2025 Sia J et al. *JAMA Network Open*.

The main scenario is shown in bold font.

© 2025 Sia J et al. *JAMA Network Open*.

Sample size	Risk length (y)	Model	Risk (%)	Power (%, e=0.65)	Power (%, e=0.60)	Power (%, e=0.55)
1000	8	B	2.5	78	64	50
1150	8	B	2.5	83	70	54
1300	8	B	2.5	92	82	68
1000	8	A	3.0	86	74	59
1000	9	B	3.0	87	75	60
**1150**	**8**	**A**	**3.0**	**92**	**82**	**68**
1150	9	B	3.0	93	83	69
1300	8	A	3.0	96	88	76
1300	9	B	3.0	93	84	69
1000	10	B	3.5	92	82	68
1150	10	B	3.5	95	87	73
1300	10	B	3.5	97	90	77
1000	9	A	3.6	93	84	70
1150	9	A	3.6	95	87	74
1300	9	A	3.6	98	93	83
1000	11	B	4.0	95	87	74
1150	11	B	4.0	98	93	82
1300	11	B	4.0	99	96	88
1000	10	A	4.3	97	90	78
1150	10	A	4.3	98	94	84
1300	10	A	4.3	99	96	88
1000	12	B	4.4	97	91	80
1150	12	B	4.4	99	94	84
1300	12	B	4.4	100	97	91
1000	11	A	4.9	98	94	84
1150	11	A	4.9	99	97	90
1300	11	A	4.9	100	98	94
1000	12	A	5.5	99	96	88
1150	12	A	5.5	100	98	92
1300	12	A	5.5	100	99	96
1000	Menopause	B	6.6	100	98	93
1150	Menopause	B	6.6	100	99	97
1300	Menopause	B	6.6	100	100	98
1000	Menopause	A	8.3	100	100	98
1150	Menopause	A	8.3	100	100	99
1300	Menopause	A	8.3	100	100	100

© 2025 Sia J et al. *JAMA Network Open*.

eTable 4. Estimated non-inferiority threshold for 90% power testing that the cancers prevented between RRES and RRSO is at least 20%. Scenarios use different follow-up length (same for all), penetrance estimates (A= Chen et al (2020), B= BOADICEA, 1980+ cohort; eTable 1), and effect sizes for RRES (e=0.65 main scenario, 0.60,0.55 as sensitivity analyses)
The main scenario is shown in bold font.

Sample size	Risk length (y)	Model	Risk RRES(%)	Risk RRSO(%)	Threshold (%, e=0.65)	Threshold (%, e=0.60)	Threshold (%, e=0.55)
1000	8	B	2.5	3.1	20	12	3
1150	8	B	2.5	3.1	23	16	9
1300	8	B	2.5	3.1	27	19	10
1000	8	A	3.0	3.9	24	16	9
1000	9	B	3.0	3.6	24	16	8
**1150**	**8**	**A**	**3.0**	**3.9**	**28**	**20**	**12**
1150	9	B	3.0	3.6	27	20	12
1300	8	A	3.0	3.9	30	22	16
1300	9	B	3.0	3.6	30	22	16
1000	10	B	3.5	4	27	20	13
1150	10	B	3.5	4	31	23	16
1300	10	B	3.5	4	32	25	19
1000	9	A	3.6	4.5	29	21	14
1150	9	A	3.6	4.5	32	24	17
1300	9	A	3.6	4.5	34	26	20
1000	11	B	4.0	4.4	31	23	17
1150	11	B	4.0	4.4	33	26	19
1300	11	B	4.0	4.4	36	28	21
1000	10	A	4.3	5	32	25	18
1150	10	A	4.3	5	36	27	21
1300	10	A	4.3	5	37	30	23
1000	12	B	4.4	4.8	34	26	18
1150	12	B	4.4	4.8	36	28	22
1300	12	B	4.4	4.8	38	31	24
1000	11	A	4.9	5.6	35	27	21

© 2025 Sia J et al. *JAMA Network Open*.

1150	11	A	4.9	5.6	37	31	24
1300	11	A	4.9	5.6	39	32	26
1000	12	A	5.5	6	37	30	23
1150	12	A	5.5	6	39	32	25
1300	12	A	5.5	6	41	34	27
1000	Menopause	B	6.6	5.7	39	33	27
1150	Menopause	B	6.6	5.7	42	35	29
1300	Menopause	B	6.6	5.7	44	37	31
1000	Menopause	A	8.3	7.1	43	36	30
1150	Menopause	A	8.3	7.1	45	38	32
1300	Menopause	A	8.3	7.1	46	40	34

eMethods. Supplementary Methods

Assumed risks

Assumptions were required on estimated risks for women aged 30 to 52y by BRCA1/2 status and age. Our primary scenario used data from a meta analysis (Chen et al, 2020).[1] This reported estimated penetrance until the end of 40y and 50y for BRCA1/2 carriers, and a chart showing a fit to risk on a continuous basis (that has been incorporated into the BRCAPRO model). However, the continuous risks were not reported. Therefore, we approximated the risk by assuming a 10y linear (increasing) hazards (eTable 1). To evaluate robustness to assumptions about expected risk, we also used BRCA1/2 penetrance assumptions obtained from the BOADICEA model,[2,3] which are lower than the meta-analysis risks (eTable 1).

Link for Source Code –

https://github.com/brentnall/protector-estimand

© 2025 Sia J et al. *JAMA Network Open*.

eFigure. One-sample analysis of DO vs no surgery

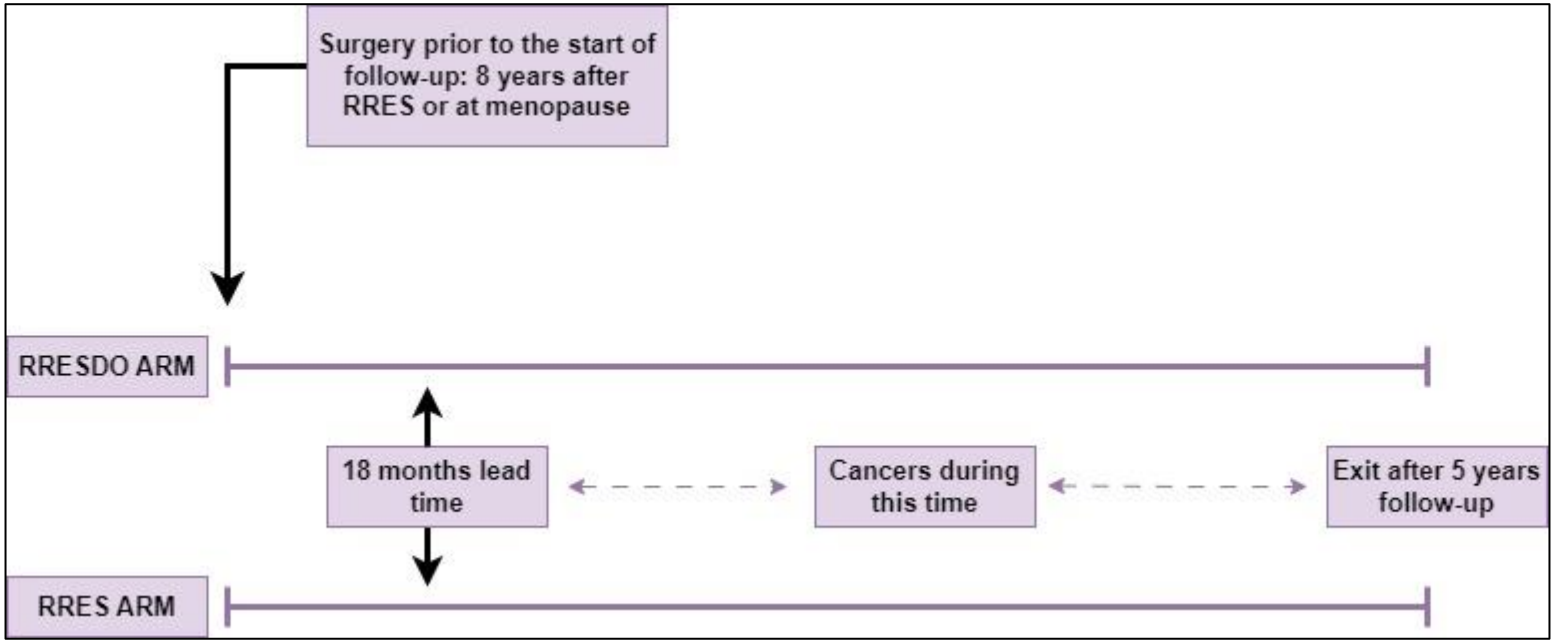

Abbreviations: RRES- Risk-reducing Early Salpingectomy; RRESDO- Risk-reducing Early Salpingectomy & Delayed Oophorectomy

© 2025 Sia J et al. *JAMA Network Open*.

eBox. Sample size assumptions

♦ The expected OC incidence in *BRCA1* and *BRCA2* PV carriers is estimated conditional on age and *BRCA1/BRCA2* status, based on data from a meta-analysis (supplementary etable-1).[1] Sensitivity analysis uses BOADICEA/CanRisk model.[2,4]

♦ The base case has an effect size of a 65% risk reduction (RR=0.35)[5] for RRES women. For sensitivity analysis we use 55% and 60% risk-reduction

♦ Risk reducing surgery will identify a small percentage of women with occult cancer, (following histology despite normal Ca125 and ultrasound scan), even if it does not prevent OC later on. We assume this is explained by OC risk within 1.5Y after surgery (lead time).

♦ If risk-reducing surgery does not prevent cancer, then the expected number of people in PROTECTOR diagnosed with OC after surgery may be estimated by summing the risk using above, from the age at 1.5Y after surgery to end of follow-up after surgery.

♦ The age and *BRCA1/BRCA2* distribution from the PROTECTOR trial for initial 889 women recruited will continue for the new study (PROTECTOR-2).[6]

♦ This estimates base case expected OC incidence of 3% for RRES and 4.4% for RRSO groups, based on 8Y follow-up. Etable-2 shows expected incidence (without surgery) for 8/10y follow-up (or to age 52y) from the two incidence assumptions.

♦ We allow for a 5% sample drop out (ie. no data at all).

♦ There is an effect-size of 96% risk-reduction (RR=0.04, i.e. from 4.4% to 0.2%) for RRSO. Thus, we assume a true ratio of ovarian cancers prevented by RRES to RRSO to be 68% (65/96).

♦ We wish to test whether RRES confers at least a 20% relative-reduction in risk (i.e. to 3*0.8 = 2.4%), otherwise findings would have limited clinical utility. This is based on our judgement but note this threshold choice (and effect-size estimate above for RRES) will drive the sample size required.

♦ The threshold for RRSO (secondary analysis) was chosen based on the sample size to achieve sufficient power for RRES, if recruitment continues as the current PROTECTOR cohort. It demonstrates the level of precision expected for this analysis and shows the study would provide useful data to help refine quantification of the effect of RRSO on risk, in combination with other data already published.

♦ Range of power estimates for a one-sample O/E test given sample size of a preventive effect at least 20%, for different analysis time follow-up lengths, penetrance estimates, and effect sizes for RRES are given in Etable-3.

© 2025 Sia J et al. *JAMA Network Open*.

~~Link for Source Code –~~

~~https://github.com/brentnall/protector-estimand~~

eReferences

1. Chen J, Bae E, Zhang L, et al. Penetrance of Breast and Ovarian Cancer in Women Who Carry a BRCA1/2 Mutation and Do Not Use Risk-Reducing Salpingo-Oophorectomy: An Updated Meta-Analysis. *JNCI Cancer Spectr*. Aug 2020;4(4):pkaa029. doi:10.1093/jncics/pkaa029
2. Lee A, Mavaddat N, Cunningham A, et al. Enhancing the BOADICEA cancer risk prediction model to incorporate new data on RAD51C, RAD51D, BARD1 updates to tumour pathology and cancer incidence. *J Med Genet*. Dec 2022;59(12):1206-1218. doi:10.1136/jmedgenet-2022-108471
3. Lee A, Yang X, Tyrer J, et al. Comprehensive epithelial tubo-ovarian cancer risk prediction model incorporating genetic and epidemiological risk factors. *J Med Genet*. Nov 29 2021;doi:10.1136/jmedgenet-2021-107904
4. Lee A, Yang X, Tyrer J, et al. Comprehensive epithelial tubo-ovarian cancer risk prediction model incorporating genetic and epidemiological risk factors. *J Med Genet*. Jul 2022;59(7):632-643. doi:10.1136/jmedgenet-2021-107904
5. Falconer H, Yin L, Gronberg H, Altman D. Ovarian cancer risk after salpingectomy: a nationwide population-based study. Research Support, Non-U.S. Gov't. *J Natl Cancer Inst*. Feb 2015;107(2)doi:10.1093/jnci/dju410
6. Gaba F, Robbani S, Singh N, et al. Preventing Ovarian Cancer through early Excision of Tubes and late Ovarian Removal (PROTECTOR): protocol for a prospective non-randomised multi-center trial. *Int J Gynecol Cancer*. Sep 8 2020;doi:10.1136/ijgc-2020-001541

© 2025 Sia J et al. *JAMA Network Open*.
