# Supplementary material for: Estimands for Clinical Effectiveness of Risk-Reducing Early Salpingectomy in Women With High Risk of Ovarian Cancer
Source: JAMA Netw Open. 2025 Sep 16;8(9):e2532195. doi: 10.1001/jamanetworkopen.2025.32195 (PMC12441877; doi:10.1001/jamanetworkopen.2025.32195)
Supplement: Supplement 2. — PROTECTOR Team Members [file jamanetwopen-e2532195-s002.pdf]

Supplemental Online Content: Nonauthor Collaborators
*First name, last name, and suffix (if applicable) are required and will appear in PubMed.

*Group Name(s): PROTECTOR team

*First Name and Middle Initial(s)	*Last Name	*Suffix (eg, Jr, III)	Academic Degrees	Institution	Location (city, state/province, country)	Role or Contribution, eg, chair, principal investigator	Group (if more than 1 Group listed in the byline) and/or Subgroup (eg, Steering Committee)
Emma	Crosbie	Prof		Manchester University NHS Foundation	United Kingdom	Local site investigator and recruitment support	
Sudha	Sundar	Prof		Sandwell and West Birmingham Hospit	United Kingdom	Local site investigator and recruitment support	
Munaza	Ahmed	Dr		North East Thames Cancer Genetics Se	United Kingdom	Genetics management MDT support	
Naveena	Singh	Prof		Barts Health NHS Trust	Deceased	Committee member	CPR Committee
Asma	Faruqi	Dr		Barts Health NHS Trust	United Kingdom	Committee member	CPR Committee
Gareth	Bryson	Dr		NHS Greater Glasgow and Clyde	United Kingdom	Committee member	CPR Committee
Gareth	Rowlands	Dr		Cardiff & Vale University Health Board	United Kingdom	Committee member	CPR Committee
Rupali	Arora	Dr		University College London Hospitals NH	United Kingdom	Committee member	CPR Committee
Giorgia	Trevisan	Dr		University College London Hospitals NH	United Kingdom	Committee member	CPR Committee
Laura	Casey	Dr		Barts Health NHS Trust	United Kingdom	Committee member	CPR Committee
Jacqueline	McDermott	Dr		Barts Health NHS Trust	United Kingdom	Committee member	CPR Committee
Thomas	Pilkington	Dr		Manchester University NHS Foundation	United Kingdom	Committee member	CPR Committee
Nataliya	Piletska	Dr		Maidstone and Tunbridge Wells NHS Tr	United Kingdom	Committee member	CPR Committee
Aarti	Sharma	Dr		Cardiff and Vale UHB	United Kingdom	Site Local Principal investigator	
Gautam	Mehra	Dr		Guy’s and St Thomas’ NHS Foundation	United Kingdom	Site Local Principal investigator	
Adam	Rosenthal	Prof		University College London Hospitals NH	United Kingdom	Site Local Principal investigator	
Ian	Harley	Dr		Belfast Health & Social Care Trust	United Kingdom	Site Local Principal investigator	
Michelle	Mackintosh	Dr		Manchester University NHS Foundation	United Kingdom	Site Local Principal investigator	
Sadaf	Ghaem-Maghami	Prof		Imperial College Healthcare NHS Trust	United Kingdom	Site Local Principal investigator	
Omer	Devaja	Prof		Maidstone and Tunbridge Wells NHS Tr	United Kingdom	Site Local Principal investigator	
Janos	Balega	Dr		Sandwell and West Birmingham Hospit	United Kingdom	Site Local Principal investigator	
Tim	Duncan	Dr		Norfolk and Norwich University Hospit	United Kingdom	Site Local Principal investigator	
Iain	Cameron	Dr		Gateshead Health NHS Foundation Tru	United Kingdom	Site Local Principal investigator	
Claire	Newton	Dr		University Hospitals Bristol NHS Found	United Kingdom	Site Local Principal investigator	
Sonali	Kaushik	Dr		Brighton and Sussex University Hospita	United Kingdom	Site Local Principal investigator	
Angela	Brady	Dr		London North West Healthcare NHS Tr	United Kingdom	Site Local Principal investigator	
Bianca	De Souza	Dr		London North West Healthcare NHS Tr	United Kingdom	Site Local Principal investigator	
Supratik	Chattopadhyay	Dr		University Hospitals of Leicester NHS T	United Kingdom	Site Local Principal investigator	
Natalia	Povolotskaya	Dr		Portsmouth Hospitals NHS Trust	United Kingdom	Site Local Principal investigator	
Rema	Iyer	Dr		East Kent Hospitals University NHS Trus	United Kingdom	Site Local Principal investigator	

Supplemental Online Content: Nonauthor Collaborators
*First name, last name, and suffix (if applicable) are required and will appear in PubMed.

*First Name and Middle Initial(s)	*Last Name	*Suffix (eg, Jr, III)	Academic Degrees	Institution	Location (city, state/province, country)	Role or Contribution, eg, chair, principal investigator	Group (if more than 1 Group listed in the byline) and/or Subgroup (eg, Steering Committee)
Lucy	Side	Dr		University Hospital Southampton NHS	United Kingdom	Site Local Principal investigator	
Katie	Snape	Dr		St George's University Hospitals NHS Fo	United Kingdom	Site Local Principal investigator	
Amal	Singh	Dr		St George's University Hospitals NHS Fo	United Kingdom	Site Local Principal investigator	
Anil	Tailor	Dr		Royal Surrey County Hospital Foundati	United Kingdom	Site Local Principal investigator	
Manon	Van Seters	Dr		Worcestershire Acute Hospital Trust	United Kingdom	Site Local Principal investigator	
Katherine	Edey	Dr		Royal Devon & Exeter NHS Foundation	United Kingdom	Site Local Principal investigator	
Sian	Taylor	Dr		Liverpool Women's Hospital NHS Foun	United Kingdom	Site Local Principal investigator	
Monika	Oktaba	Dr		Liverpool Women's Hospital NHS Foun	United Kingdom	Site Local Principal investigator	
Suma	Kodiathodi	Dr		North Tees and Hartlepool NHS Fo	United Kingdom	Site Local Principal investigator	
Partha	Sengupta	Dr		County Durham and Darlington NHS	United Kingdom	Site Local Principal investigator	
Scott	Fegan	Dr		NHS Lothian	United Kingdom	Site Local Principal investigator	
Karin	Williamson	Dr		Nottingham University Hospitals NH	United Kingdom	Site Local Principal investigator	
Andrew	Phillips	Dr		University Hospitals of Derby and B	United Kingdom	Site Local Principal investigator	
Mark	Willett	Dr		East Lancashire Hospitals NHS Tru	United Kingdom	Site Local Principal investigator	
Tony	Chalhoub	Dr		The Newcastle upon Tyne Hospitals NH	United Kingdom	Site Local Principal investigator	
Rachel	O'Donnell	Dr		The Newcastle upon Tyne Hospitals NH	United Kingdom	Site Local Principal investigator	
Sanjay	Rao	Dr		South Tees Hospitals NHS Foundation T	United Kingdom	Site Local Principal investigator	
Nicholas	Matthews	Dr		South Tyneside and Sunderland NHS Fo	United Kingdom	Site Local Principal investigator	
Beena	Abdul	Dr		Oxford University Hospitals NHS Trust	United Kingdom	Site Local Principal investigator	
Chellappah	Gnanachandran	Dr		Northampton General Hospitals NHS Tr	United Kingdom	Site Local Principal investigator	
Claire	Park	Dr		Royal United Hospitals Bath NHS Found	United Kingdom	Site Local Principal investigator	
Jane	Borley	Dr		Royal Cornwall Hospitals NHS Trust	United Kingdom	Site Local Principal investigator	
Richard	Hutson	Dr		Leeds Teaching Hospitals NHS Trust	United Kingdom	Site Local Principal investigator	
John	Dalton	Dr		Leeds Teaching Hospitals NHS Trust	United Kingdom	Site Local Principal investigator	
Richard	Peevor	Dr		Betsi Cadwaladr University Health Boar	United Kingdom	Site Local Principal investigator	
Atiyah	Kamran	Dr		NHS Grampian	United Kingdom	Site Local Principal investigator	
Mahalakshmi	Gurumurthy	Dr		NHS Grampian	United Kingdom	Site Local Principal investigator	
Kalpana	Ragupathy	Dr		NHS Tayside	United Kingdom	Site Local Principal investigator	
Helen	Bolton	Dr		Cambridge University Hospitals NHS Fo	United Kingdom	Site Local Principal investigator	
Jenifer	Sassarini	Dr		NHS Greater Glasgow and Clyde	United Kingdom	Site Local Principal investigator	
Nicholas	Matthews	Dr		South Tyneside and Sunderland NHS Fo	United Kingdom	Site Local Principal investigator	
Mithila	Prasad	Dr		East Lancashire Hospitals NHS Trust	United Kingdom	Site Associate principal investigator	
Monica	Tryczynska	Dr		Cardiff and Vale UHB	United Kingdom	Site Associate principal investigator	
Roula	Elboraei	Dr		Cardiff and Vale UHB	United Kingdom	Site Associate principal investigator	

Supplemental Online Content: Nonauthor Collaborators
*First name, last name, and suffix (if applicable) are required and will appear in PubMed.

***First Name and Middle Initial(s)**	***Last Name**	***Suffix (eg, Jr, III)**	Academic Degrees	Institution	Location (city, state/province, country)	Role or Contribution, eg, chair, principal investigator	Group (if more than 1 Group listed in the byline) and/or Subgroup (eg, Steering Committee)
Gisela	Reig	Dr		NHS Tayside	United Kingdom	Site Associate principal investigator	
Kelly	Kohut	Dr		St George’s University Hospitals NHS Fo	United Kingdom	Site Associate principal investigator	
Victor	Ohwo	Dr		Gateshead Health NHS Foundation Tru	United Kingdom	Site Associate principal investigator	
Victoria	Barker	Dr		University College London Hospitals NH	United Kingdom	Site Associate principal investigator	
Halimah	Alazzani	Dr		University College London Hospitals NH	United Kingdom	Site Associate principal investigator	
Neil	Ryan	Dr		Royal Infirmary of Edinburgh NHS Lothi	United Kingdom	Site Associate principal investigator	
Nazish	Zulfiqar	Dr		Portsmouth Hospitals NHS Trust	United Kingdom	Site Associate principal investigator	
Mohamed	Abdelaziz	Dr		Nottingham University Hospitals NHS T	United Kingdom	Site Associate principal investigator	
Georgina	McArdle	Dr		Gateshead Health NHS Foundation Tru	United Kingdom	Site Associate principal investigator	
Hyunsu	Doh	Dr		Gateshead Health NHS Foundation Tru	United Kingdom	Site Associate principal investigator	
Kathryn	Baxter	Dr		Manchester University NHS Foundation	United Kingdom	Site Associate principal investigator	
Irene	Ray	Dr		Brighton and Sussex University Hospita	United Kingdom	Site Associate principal investigator	
Thu Thu	Khaing	Dr		Portsmouth Hospitals NHS Trust	United Kingdom	Site Associate principal investigator	
Maria	Marks	Dr		Norfolk and Norwich University Hospita	United Kingdom	Site Associate principal investigator	
Helena	Misiura	Dr		East Lancashire Hospitals NHS Trust	United Kingdom	Site Associate principal investigator	
