# Supplementary material for: Estimands for Clinical Effectiveness of Risk-Reducing Early Salpingectomy in Women With High Risk of Ovarian Cancer
Source: JAMA Netw Open. 2025 Sep 16;8(9):e2532195. doi: 10.1001/jamanetworkopen.2025.32195 (PMC12441877; doi:10.1001/jamanetworkopen.2025.32195)
Supplement: Supplement 3. — Data Sharing Statement [file jamanetwopen-e2532195-s003.pdf]

Data Sharing Statement

Sia. Estimands for Clinical Effectiveness of Risk-Reducing Early Salpingectomy in High-Risk Women. *JAMA Netw Open*. Published September 16, 2025.
doi:10.1001/jamanetworkopen.2025.32195

Data
Data available: No

Additional Information
Explanation for why data not available: Summary statistics of the data underpinning the analysis including expected risks have been provided in the manuscript. As the individual patient level data used is from an ongoing prospective trial which has not yet been published, this cannot be currently shared till the trial has published. We have provided the link for the source code for analysis in the supplement
